# The prevalence of* Pseudomonas aeruginosa* and multidrug resistant *Pseudomonas aeruginosa* in healthy captive ophidian

**DOI:** 10.7717/peerj.6706

**Published:** 2019-04-12

**Authors:** Andrea Sala, Francesco Di Ianni, Igor Pelizzone, Mara Bertocchi, Davide Santospirito, Francesco Rogato, Sara Flisi, Costanza Spadini, Tiziano Iemmi, Emanuele Moggia, Enrico Parmigiani, Sandro Cavirani, Simone Taddei, Clotilde S. Cabassi

**Affiliations:** 1 Department of Veterinary Science, University of Parma, Parma, Italy; 2 Veterinary Practitioner DVM, Reggio Emilia, Italy; 3 Azienda Unità Sanitaria Locale di Reggio Emilia, Reggio Emilia, Italy; 4 Veterinary Practitioner DVM, Ringwood, England; 5 Veterinary Practitioner DVM, Chiavari, Italy

**Keywords:** Pseudomonas aeruginosa, Multidrug resistant, Prevalence, Ophidians, Snakes, Antibiotic, Antimicrobial, Captive, Farming conditions

## Abstract

**Background:**

Snakes are globally considered as pet animals, and millions of ophidians are bred in captivity. *Pseudomonas aeruginosa* is a ubiquitous Gram-negative bacterium that can act as an opportunistic pathogen of man and animals and is frequently present in the oral and cloacal microbiota of healthy ophidians. It can cause severe clinical diseases and often shows antibiotic resistance. The aim of this study was to evaluate the prevalence and antibiotic resistance profiles of *P. aeruginosa* isolated from the cloacal microbiota of a large population sample of healthy captive ophidians and to evaluate the statistical associations with farming conditions.

**Methods:**

A total of 419 cloacal swabs were collected from snakes belonging to the Boidae (*n* = 45), Colubridae (*n* = 48) and Pythonidae (*n* = 326) families and inoculated onto complete culture media. Food, water and bedding samples were also analyzed. The antimicrobial susceptibility of *P. aeruginosa* isolates was evaluated through the Kirby-Bauer agar diffusion test. Statistical analyses were performed with the chi-square test.

**Results:**

The prevalence of *P. aeruginosa* was 59.9%, and 35.5% of these strains were multidrug resistant (MDR). The prevalence of MDR *P. aeruginosa* was significantly higher in adult samples than in young samples, and widespread resistance to Cephalosporins, Polymyxins and Sulfonamides was observed. Statistically significant differences in the prevalence of *P. aeruginosa* were observed depending on the farm size and snake family*.* Feeding thawed prey was associated with a higher *P. aeruginosa* and MDR *P. aeruginosa* prevalence. Moreover, snakes fed home-raised prey had a significantly higher MDR *P. aeruginosa* prevalence than snakes fed commercially available feed. Less frequent terrarium cleaning was associated with a higher MDR *P. aeruginosa* prevalence. On the other hand, snake reproductive status was not significantly associated with *P. aeruginosa* or MDR *P. aeruginosa* prevalence. All food, water and bedding samples were negative for *P. aeruginosa* presence.

**Discussion:**

The overall *P. aeruginosa* prevalence found in this study was lower than that found by other authors, but a high proportion of the isolates were MDR. This study highlighted the presence of constitutive (such as age and taxonomic family) and managerial (farm size, cleaning cycle frequency and food type) factors associated with *P. aeruginosa* and/or MDR *P. aeruginosa* prevalence. Good breeding management and proper antibiotic treatment of *P. aeruginosa* infections could help reduce the presence of *P. aeruginosa* and MDR *P. aeruginosa* in the gut microbiota of snakes and consequently reduce the risk to public health.

## Introduction

The captive breeding of reptiles has expanded considerably in recent years. Currently, many species of snakes are raised as pet animals, and good knowledge of their physiological and behavioral needs allows for proper management in captivity, where the goal is to create a domestic habitat similar to that of their origin (Stahl, 2002; Mitchell, 2004). The specific environmental conditions of the terrarium, such as temperature, humidity, aeration, moisture and organic substances, are favorable growth factors for commensal microorganisms and opportunistic pathogens (Ebani & Fratini, 2005; Romero et al., 2015). Snakes could become spreaders of potentially pathogenic bacteria, which can represent a serious threat to themselves and humans (Goldstein et al., 1981; Ebani & Fratini, 2005; Romero et al., 2015). One of the most common bacteria found in snakes’ environments is *Pseudomonas aeruginosa*. This ubiquitous Gram-negative bacterium, widely considered an opportunistic pathogen for man and animals (Lyczak, Cannon & Pier, 2000; Walker et al., 2002; Rubin et al., 2008), can be detected inside the oral and cloacal microbiota of healthy ophidians and is more frequently found in captive snakes than in wild snakes (Blaylock, 2001; Colinon et al., 2010). In snakes subjected to stressful or immunosuppressive conditions, *P. aeruginosa* can cause severe local and even systemic infections (Draper, Walker & Lawler, 1981; Paré et al., 2006; Jacobson, 2007). Unfortunately, the treatment of *P. aeruginosa* infections is often difficult due to the high occurrence of antibiotic resistance to different antibiotic classes (Hancock & Speert, 2000; Breidenstein, de la Fuente-Núñez & Hancock, 2011). To evaluate the prevalence and antibiotic resistance profiles of *P. aeruginosa* in healthy captive ophidians, we examined a large number of cloacal swabs from animals bred in Italian snake farms. Moreover, to point out potential risk factors, the prevalence of *P. aeruginosa* and multidrug resistant (MDR) *P. aeruginosa* in different farming conditions was evaluated. In addition, environmental samples were tested to identify possible sources of *P. aeruginosa* and MDR *P. aeruginosa*.

## Materials & Methods

A total of 419 cloacal swabs were collected from healthy ophidians belonging to species commonly raised as pet animals. The collection of swabs was conducted in compliance with national (Decreto Legislativo n. 26, 4 Marzo 2014) and European (Directive 2010/63/EU) laws and policies. The present project was approved by the Ethical Committee of the University of Parma (Organismo Preposto al Benessere degli Animali - prot. n. 251/OPBA/2017). All the examined snakes belonged to either the Boidae (*n* = 45), Colubridae (*n* = 48) and Pythonidae (*n* = 326) families*.* The most frequently encountered species was *Python regius* (*n* = 318). Samples were collected from animals belonging to 15 different snake farms, located mainly in Northern Italy. All the animals were captive-born. Based on the number of snakes bred, the farms were classified as large (more than 50 animals), medium (from 11 to 50 animals) and small (10 or fewer animals) in size. For each animal, an anamnestic form was completed to obtain information regarding origin, health status, medical history and farming conditions. Among these parameters, all those involved in the maintenance of a correct microenvironment (e.g., temperature, lighting, humidity, ventilation, presence of water, and feeding) were considered. The animals’ reproductive status was also evaluated. Breeders gave information about the animals’ reproductive status for only 230 of the 419 samples; statistical analyses were performed based on the collected information. Statistical analyses were performed using the chi-square test.

The manual restraint and sampling procedures were fast and minimally invasive for these animals. Cloacal swabs in Amies transport medium were kept at 4 °C and transported to the laboratory within 24 hours. The swabs were plated onto MacConkey agar (DIFCO) and Columbia blood agar with 5% of bovine erythrocytes and incubated aerobically for 24 h at 37 °C. *P. aeruginosa* identification was based on colony morphology, the presence of hemolysis, and the oxidase reaction and confirmed with the API 20 NE biochemical test system (bioMérieux, Marcy l’Etoile, France) (Markey et al., 2013). The antimicrobial susceptibility for each bacterial strain was evaluated through the Kirby-Bauer agar diffusion test. The tested antibiotics (Table 1) included those commonly used in ophidians and a larger panel of antibiotics specific for *P. aeruginosa*, as indicated by Magiorakos et al. (2012). These antibiotics, belonging to different chemical classes, are useful for the classification of all *P. aeruginosa* isolates as MDR, extensively drug resistant (XDR) or pandrug resistant (PDR) strains. A strain is considered MDR when it is resistant to one or more antibiotics in at least three or more antibiotic classes, XDR when it is resistant to at least one agent in all but two or fewer antimicrobial classes and PDR when no susceptibility is detected towards all tested antimicrobial agents.

**Table 1:** Percentages of *P. aeruginosa* isolates resistant to the different antibiotics.

**Antibiotic classes**	**Antibiotic**	**Percentage of resistant*P. aeruginosa*strains**
**Aminoglycosides**	Amikacin (30 µg)	2%
	Gentamicin (10 µg)	35%
	Tobramycin (10 µg)	9%,
**Carbapenems**	Imipenem (10 µg)	0%
**Cephalosporins**	Ceftazidime (30 µg)	1%
	Cefovecin (30 µg)	100%
	Cephazolin (30 µg)	100%
**Fluoroquinolones**	Ciprofloxacin (5 µg)	1%
	Enrofloxacin (5 µg)	10%
	Marbofloxacin (5 µg)	1%
**Penicillins and β-lactamase inhibitors**	Piperacillin + Tazobactam (100 + 10 µg)	2%
**Tetracyclines**	Doxycycline (30 µg)	9%
**Monobactams**	Aztreonam (30 µg)	2%
**Phosphonic acids**	Phosphomicin (200 µg)	8%
**Polymyxins**	Polymyxin B (300 U)	95%
	Colistin (10 µg)	94%
**Sulfonamides**	Sulfamethoxazole (25 µg)	91%
**Phenicols**	Tiamphenicol (30 µg)	64%

The presence of *P. aeruginosa* in food was investigated by testing 26 samples of frozen food (rats), and 19 fecal samples from living prey, collected in five different farms. Moreover, six water samples from six different farms and 2 fresh bedding samples from two different farms were analyzed for the presence of *P. aeruginosa*. Water samples (100 ml) were collected in duplicate in presence of 0.1 ml of sterile 10% sodium thiosulfate, kept cool and analyzed within 6 h. Each water sample was filtered through a 25 mm diameter, 0.3 µm pore size, sterile, mixed cellulose ester membrane filter (Merck Millipore, Burlington, MA, USA). The filters were then immediately placed on McConkey and blood agar media and incubated at 37 °C for 24 h. Each bedding sample was collected by sampling different parts of the container and kept cool until it was referred to the lab, where it was mix thoroughly, with a 1:10 w/v dilution ratio, with sterile 1% buffered peptone water. The sample was allowed to sit for 30 to 60 min at room temperature with frequent shaking, then it was streaked on McConkey and blood agar and incubated at 37 °C for 24 h. One milliliter of the sample was also added to 9 ml of BHI and incubated at 37 °C for 24 h. In case of *P. aeruginosa* negative McConkey and blood agar, BHI was subcultured on the same solid media.

## Results

*P. aeruginosa* was isolated from 251 (59.9%) out of 419 total examined samples. Among the 251 *P*. *aeruginosa* isolates, 89 (35.5%) were MDR*.* None of the 251 *P. aeruginosa* strains were XDR or PDR. Resistance against cefovecin and cephazolin (third-generation and first-generation cephalosporins, respectively), polymyxin B and colistin (polymyxins) and sulfamethoxazole (sulfonamides) was observed more frequently (Table 1).

The prevalence of *P. aeruginosa* was 54.4% (155/285) for animals from large farms, 73% (65/89) for animals from medium farms and 68.9% (31/45) for animals from small farms. These differences were statistically significant (*P* = 0.003). The prevalence of the MDR *P. aeruginosa* strains among *P. aeruginosa* isolates was 32.9% (51/155) for animals from large farms, 38.5% (25/65) for animals from medium farms and 41.9% (13/31) for animals from small farms. In this case, the differences were not statistically significant (*P* = 0.531).

*P. aeruginosa* was isolated from 218 out of 373 adult snakes (58.4%) and from 33 out of 46 juvenile snakes (71.7%). Differences between *P. aeruginosa* prevalence in adults and juveniles were not statistically significant (*P* = 0.083). Approximately 12.1% (4/33) of the isolates from juvenile snakes and 39% (85/218) of the isolates from adult snakes were MDR. In this case, the difference was statistically significant (*P* = 0.003).

Most of the subjects belonged to the Pythonidae family. In this family, the overall *P. aeruginosa* prevalence was 60.1% (196/326), while it was 77.8% (35/45) in Boidae and 41.7% (20/48) in Colubridae. These differences were statistically significant (*P* = 0.002). The prevalence of MDR *P. aeruginosa* strains among *P. aeruginosa* isolates was 31.6% (62/196) in Pythonidae, 48.6% (17/35) in Boidae and 50% (10/20) in Colubridae. In this case, differences were not statistically significant (*P* = 0.057).

The prevalence of *P. aeruginosa* was 50% (34/68) for animals in an active state of reproduction (gravid females or soon after spawning), of which 26.5% (9/34) were MDR strains. In snakes not in reproduction, the *P. aeruginosa* prevalence was 59.9% (97/162), of which 24.7% (24/97) were MDR strains. The observed differences in *P. aeruginosa* prevalence between these groups were not statistically significant (*P* = 0.167), nor were the differences in MDR *P. aeruginosa* prevalence (*P* = 0.842).

In Table 2, the prevalence of *P. aeruginosa* related to the type of feed given to animals is reported. Differences in the prevalence of *P. aeruginosa* and MDR *P. aeruginosa* between animals fed living or freshly dead prey and those fed thawed food were statistically significant (*P* = 0.016 and *P* = 0.007, respectively).

**Table 2:** Prevalence of *P. aeruginosa* in relation to the type of food (living/freshly death or thawed preys).

	**Number of subjects**	***P. aeruginosa*positive samples**	**MDR** ***P. aeruginosa***^a^
**Living/freshly death**	342	196 (57.3%)	62 (31.6%)
**Thawed**	55	41 (74.5%)	22 (53.7%)
**Both type**	17	11 (64.7%)	4 (36.4%)
N.D.^*^	5	3 (60%)	1 (33.3%)
**Total**	419	251 (59.9%)	89 (35.5%)

**Notes.**

a Reported percentages are referred to the total of *P. aeruginosa* positive samples.

* Not declared by breeders.

The prevalence of *P. aeruginosa* and MDR *P. aeruginosa* was evaluated in relation to the origin of the administered prey: raised at home (pinkies, rats, guinea pigs, rabbits, mice) or purchased. In snakes fed with home-raised food, the *P. aeruginosa* prevalence was 61.5% (110/179), while it was 66.2% (92/139) in snakes fed purchased food. No significant differences were observed in this case (*P* = 0.384). The prevalence of MDR *P. aeruginosa* was 28.2% (31/110) in animals fed home-raised food and 43.5% (40/92) in animals fed purchased food. The difference in the prevalence of MDR *P. aeruginosa* in the commercial food group compared to the home-raised food group was statistically significant (*P* = 0.023).

The cleaning cycle of terrariums was also investigated (Table 3). Differences in *P. aeruginosa* prevalence among animals from terrariums with different cleaning cycles were not statistically significant (*P* = 0.075). However, regarding the cleaning cycles, statistical significance was observed for differences in the prevalence of MDR *P. aeruginosa* among *P. aeruginosa-* positive samples (*P* = 0.009).

**Table 3:** Prevalence of *P. aeruginosa* in relation to the frequency of terrarium cleaning.

		**Number of subjects**	***P. aeruginosa*positive samples**	**MDR** ***P.aeruginosa***^a^
**Cleaning cycle frequency**	**Weekly**	277	159 (57.4%)	44 (27.7%)
	**Twice a month**	112	74 (66.1%)	33 (44.6%)
	**Monthly**	12	10 (83.3%)	6 (60%)
	N.D.^*^	18	8 (44.4%)	6 (75%)
	**Total**	419	251 (59.9%)	89 (35.5%)

**Notes.**

a Reported percentages are referred to the total of *P. aeruginosa* isolates.

* Not declared by breeders.

All food, water and bedding samples were negative for *P. aeruginosa* presence.

## Discussion

The occurrence of antimicrobial resistance in bacteria, such as *P. aeruginosa*, has become a global emergency, and it is one of the major challenges that humanity will face in the future (Fowler, Walker & Davies, 2014). Antimicrobial-resistant bacteria are increasingly involved in infections that may affect different animal species. Furthermore, it is not unusual to find *P. aeruginosa* strains carrying resistant genes within the microbiota of different organisms (Sørum & Sunde, 2001; Szmolka & Nagy, 2013). *P. aeruginosa* is an opportunistic pathogen that is part of the normal gut microbiota of many vertebrates, including snakes. In the presence of debilitating factors for ophidians, this bacterium can express its pathogenicity, resulting in secondary infections involving a wide range of tissues (Schumacher, 2006; Chinnadurai & DeVoe, 2009). *P. aeruginosa*’s ability to easily develop antibiotic resistance (Breidenstein, de la Fuente-Núñez & Hancock, 2011), coupled with its extensive spread within terrariums, similar to what occurs for parasites (Ippen & Zwart, 1996; Raś-Noryńska & Sokół, R, 2015), makes *P. aeruginosa* a risk factor for ophidians and people who handle these animals. Currently, there are few reports of *P. aeruginosa* infections in reptiles. This study involved a large number of animals and showed that the fecal carriage of *P. aeruginosa* frequently occurs among healthy captive snakes. Therefore, *P. aeruginosa* can be considered part of the gut microbiota of healthy ophidians. However, the overall *P. aeruginosa* prevalence found here (59.9%) was lower than expected based on other studies performed on captive snakes (Colinon et al., 2010; Foti et al., 2013; Dipineto et al., 2014). Eighty-nine (35.5%) of the *P. aeruginosa* isolates were MDR. The high rate of MDR strains could be related to the occurrence of antibiotic-resistant determinants in *P. aeruginosa* isolated from anthropic environments (Igbinosa et al., 2012; Kuczynski, 2016).

Our data suggest that the probability of harboring *P. aeruginosa* could be linked to the taxonomic family of the snake. However, the same association was not observed between the taxonomic family and the prevalence of MDR *P. aeruginosa*. The *P. aeruginosa* prevalence in adults and juveniles was greater than 50%, and the differences were not statistically significant between age groups. However, the probability of finding MDR *P. aeruginosa* strains in adults was significantly higher than in juveniles. This could be linked to the higher probability that adults will be exposed to external MDR *P. aeruginosa* strains, due to their longer lifespan, but probably not to a greater exposure of adults to antibiotics. In fact, the use of antibiotics in snake farms is infrequent and only three of the analyzed subjects, all from the same farm, were previously treated, specifically with marbofloxacin.

Many authors have indicated that stress is a condition that predisposes the infected host to the development of *P. aeruginosa-*derived diseases (Ebani & Fratini, 2005; Schumacher, 2006). In ophidians, an active state of reproduction is considered a very stressful condition (Moore & Jessop, 2003) that is characterized by a lower than optimal temperature (Mathies & Miller, 2003). Moreover, direct contact during mating could favor the transmission of cloacal *P. aeruginosa*. Nevertheless, no significant differences in the prevalence of *P. aeruginosa* and MDR *P. aeruginosa* were observed between animals in active and non-active states of reproduction. In humans, the transmission of *P. aeruginosa* from mothers to *their* offspring can occur, especially in case of preterm premature rupture of membranes (Casetta et al., 2003; Cortese et al., 2016). However, to our knowledge, there are no reports regarding the vertical transmission of *P. aeruginosa* in snakes. In this study, the vertical transmission between animals in breeding condition and their offspring was not evaluated. However, in general, horizontal transmission play a predominant role in the diffusion of *P. aeruginosa* and probably we can assume the same in snakes.

Regarding animal feeding, there is a higher probability of *P. aeruginosa* harboring in animals fed thawed prey. Likewise, animals fed thawed prey showed a higher prevalence of MDR *P*. *aeruginosa*. Almost all the thawed prey were purchased, while most of the living prey were home-raised. Moreover, the purchased frozen food was distributed by a single supplier for all farms. Pseudomonadaceae can survive at freezing temperature (Lu et al., 2011; Chauhan et al., 2015). Therefore, our initial hypothesis was that *P. aeruginosa* could be selected compared to other freezing-sensitive gut microbiota species and replicate in defrosted food. Moreover, *P. aeruginosa* at freezing temperatures undergoes several changes to its constitutive components (e.g., the cell wall and membrane) that regulate the passage of many molecules, including antibiotics (Lu et al., 2011) and this could alter the response of *P. aeruginosa* to antimicrobials. However, we have found negative results for *P. aeruginosa* isolation from food, both frozen and fresh, and this does not allow us to establish the cause of the obtained result. Nevertheless, a role of food in the transmission of *P. aeruginosa* cannot be excluded. Indeed, *P. aeruginosa* can easily thrive within snakes and they can be considered reservoirs of the bacterium (Colinon et al., 2010). This means that it is not possible to establish since the animals were infected and previous batches of food from the supplier could have given different results. The fact that the examined farms are open farms, in which the entry and exit of animals occur, combined with the fact that these animals are not checked before entering in the farm (only sometimes they are placed in quarantine) and that the infection is asymptomatic, are further elements that makes it difficult to establish the origin of *P. aeruginosa* found in snakes. Horizontal transmission of *P. aeruginosa* to human patients through the hands of clinical staff and bacterial transmission due to handling of snakes were suggested (Deplano et al., 2005; Bemis et al., 2007). Therefore, also in light of the negative results on food, water and bedding samples, the direct or indirect transmission of *P. aeruginosa* and the movement of animals may have played an important role for the spread of the bacterium.

Farming conditions are essential for captive ophidians’ health. The lower prevalence of *P. aeruginosa* found in large farms compared to medium and small farms could be due to higher hygienic standards, in addition to the fact that large farms are usually closed herds. As shown in Table 3, frequent terrarium cleaning reduced the percentage of *P. aeruginosa-* positive samples from 83.3% for monthly cleaning to 57.4% for weekly cleaning. However, differences in *P. aeruginosa* prevalence found for the different cleaning cycles were not statistically significant. However, the proportion of MDR *P. aeruginosa* (60% for monthly cleaning and 27.7% for weekly cleaning) was significantly different depending on the frequency of cleaning. For bacteria in the terrarium, less frequent cleaning operations could increase the probability of coming into contact with bacterial strains carrying MDR genes (Soda et al., 2008).

Reptiles are considered reservoirs of different zoonotic microorganisms (Goldstein et al., 1981; Ebani & Fratini, 2005; Martins et al., 2017). Among them, *P. aeruginosa* was found in rattlesnakes venom (Goldstein et al., 1979) and isolated from infected wounds of humans caused by snake bite (Garg et al., 2009). Moreover, *P. aeruginosa* cross-contamination between captive snakes and owners was reported (Colinon et al., 2010). The emergence and spread of drugs resistant *P. aeruginosa* strains, whose increasing rates are a worldwide public health problem, is frequently ascribed to patient-to-patient transmission of resistant strains and may be associated with previous antibiotic exposure (Raman et al., 2018). In the present study, some risk factors associated with the presence of MDR strains were highlighted, even if it was not possible to establish the origin of those strains, and animal-to-animal transmission may have played an important role. On the other hand, exposure to antibiotics, rare in the examined farms, may not have been a main factor. However, little information is available on the transmission of *P. aeruginosa* between captive ophidians and humans and further studies to establish their zoonotic potential are needed.

## Conclusions

This study included healthy captive snakes bred in highly controlled environmental conditions; these conditions facilitated the evaluation of the association between the prevalence of *P. aeruginosa* and farming conditions. The study highlighted the presence of constitutive (such as age and taxonomic family) and managerial (frequency of cleaning cycle and type of food) factors associated with *P. aeruginosa* and/or MDR *P. aeruginosa* prevalence. Antibiotic resistance is a complex phenomenon, and the details of its mechanism are often reduced to the incorrect use of antibiotics. *P. aeruginosa* showed an excellent ability to develop resistance against a wide range of antimicrobials through various molecular mechanisms (Moradali, Ghods & Rehm, 2017). Therefore, in view of the capability of *P. aeruginosa* to become resilient during pathogenesis to withstand antibacterial treatment, a management program is still required to fight infections (Moradali, Ghods & Rehm, 2017). The data obtained suggest that the prevalence of MDR *P. aeruginosa* strains could be influenced and partially limited through managerial choices. Regarding the treatment of *P. aeruginosa* infections, the wide resistance to cephalosporins, polymyxins and sulfonamides found here suggests that other antibiotic classes should be chosen for routine clinical practice. However, bacteriological examinations and antimicrobial sensitivity tests are always recommended before any antibiotic treatment.

In conclusion, good breeding management could help to reduce the presence of *P. aeruginosa* and MDR *P. aeruginosa* in the gut microbiota of these animals and, consequently, in the farm environment. This could also be beneficial for reducing the risk of *P. aeruginosa* infections in other animals and humans.

##  Supplemental Information

10.7717/peerj.6706/supp-1 Data S1 Raw data

## Additional Information and Declarations

### Data Availability

The following information was supplied regarding data availability: The raw data is available in the ‘Results’ section and in Data S1.
